# Tannic acid coating gauze immobilized with thrombin with ultra-high coagulation activity and antimicrobial property for uncontrollable hemorrhage

**DOI:** 10.1038/s41598-024-67049-y

**Published:** 2024-07-12

**Authors:** Jian Ye, Chenyang Shi, Jian Lan, Qingqing Chen, Qin Si, Panpan Xu, Xijiang Zhang, Cheng Zheng

**Affiliations:** 1 Department of Critical Care Medicine, Municipal Hospital Affiliated to Taizhou University, Zhejiang, 318000 China; 2 Department of Critical Care Medicine, Taizhou Hospital of Zhejiang Province Affiliated to Wenzhou Medical University, Zhejiang, 318000 China

**Keywords:** Prehospital care, Hemostasis, Gauze, Tannic acid, Thrombin, Translational research, Trauma

## Abstract

Rapid and safe hemostasis is crucial for the survival of bleeding patients in prehospital care. It is urgent to develop high performance hemostatic material to control the massive hemorrhage in the military field and accidental trauma. In this work, an efficient protein hemostat of thrombin was immobilized onto commercial gauze, which was mediated by self-polymerization and anchoring of tannic acid (TA). Through TA treatment, the efficient immobilization of thrombin was achieved, preserving both the biological activity of thrombin and the physical properties of the dressing, including absorbency, breathability, and mechanical performance. Moreover, in the presence of TA coating and thrombin, Gau@TA/Thr could obviously shortened clotting time and enriched blood components such as plasma proteins, platelets, and red blood cells, thereby exhibiting an enhanced in vitro coagulation effect. In SD rat liver volume defect and artery transection hemorrhage models, Gau@TA/Thr still had outstanding hemostatic performance. Besides, the Gau@TA/Thr gauze had inherent antibacterial property and demonstrated excellent biocompatibility. All results suggested that Gau@TA/Thr would be a potential candidate for treating uncontrollable hemorrhage in prehospital care.

## Introduction

Acute bleeding is one of the leading causes of death worldwide each year, especially in the uncontrolled major bleeding that occurs during wars, disasters, and emergency rescue operations^1,2^. Effective prehospital bleeding control is of paramount importance^3,4^. In general, the rapid and effective use of hemostatic materials in the early stage of bleeding can prolong the rescue time and reduce the high mortality rate caused by excessive bleeding^5,6^. Additionally, prolonged exposure of the wound to air makes it susceptible to bacterial infection. Therefore, the design of high-performance and safe hemostatic materials has always been a hot topic in academic research and industrial innovation.

Various hemostatic materials, including gauze, powder, and tissue adhesive, have been designed and developed to control hemorrhage^7–10^. Previous studies have shown that commercial CELOX™, HemCon^®^, and QuikClot^®^ have a significant hemostatic effect in the emergency treatment of bleeding in wars and accidents^11,12^. However, these materials lack sufficient procoagulant ability, leading to limited hemostatic effectiveness. For example, chitosan-based hemostatic powder and hemostatic gauze typically accelerate blood clotting by interacting with platelets in the blood^13–15^. Silicon-based mineral hemostatic material (including kaolin, montmorillonite and halloysite) accelerates the initiation of the coagulation process by activating the Factor XII^16–18^. The promotion of coagulation mechanisms can indeed shorten the coagulation process, but it still relies on the body's own coagulation system, known as the coagulation cascade reaction. As a result, this approach still cannot meet the needs of emergency hemostasis.

As we all know, thrombin, also known as coagulation factor II, can bypass most of the processes of the coagulation cascade and directly catalyze fibrinogen conversion into fibrin, forming a stable fibrin clot to promote hemostasis^19^. In addition, thrombin has no adverse effects on foreign body reactions. In clinical practice, the physiological saline solution of thrombin is often used to treat visceral bleeding as well as blood vessels that are difficult to ligate for hemostasis^20,21^. However, excessive use of thrombin, or thrombin entering open wounds and subsequently entering the bloodstream would result in hypercoagulability, even leading to thrombus. For safe and efficient utilize of thrombin, it is commonly loaded or immobilized onto a polymer substrate. For instance, Shi et al. prepared CaCO_3_-based microsphere with a low concentration of thrombin on it^20^. Li et al. developed a thrombin/cross-linked grapheme sponge in which thrombin was absorbed into the inner porous sponge to avoid burst release of thrombin^22^. However, they are either complex to prepare or expensive and inconvenient, and these materials are not suitable for controlling major bleeding in the process of first aid. To date, it is urgently needed to find an expedient method to prepare the thrombin-based material with high hemostatic performance, antibacterial property, and bio-safety for prehospital bleeding control.

In this study, we prepared a thrombin-based composite gauze (Gau@TA/Thr) which immobilize thrombin onto the surface of cellulose gauze by utilizing the phenolic hydroxyl group and hydrophobic segments of tannic acid (TA)^23,24^. After a simple treatment with TA and loading of thrombin, the Gau@TA/Thr gauze not only retains its original liquid absorption, air permeability, and mechanical properties, but also exhibits extremely high pro-coagulant activity. In different massive hemorrhage models of SD rats, Gau@TA/Thr gauze also demonstrated a remarkably high hemostatic effect. Besides, the results indicated that Gau@TA/Thr gauze had inherent antibacterial property excellent biocompatibility. As a result, the thrombin-based composite gauze might have great application potential in prehospital massive hemorrhage control.

## Materials and methods

### Materials

Cellulose gauze (Gau) was purchased from Zhende Medical Supplies Co., Ltd. Tannic acid (TA) was purchased from Shanghai Aladdin biochemical technology co. LTD. China. Thrombin (2000 U/mg) from bovine plasma was purchased from Shanghai Maclin Biochemical Technology Co., LTD. Thrombin chromogenic substrate (S-2238) was purchased from HYPHEN BioMed. Dulbecco's modified eagle medium (DMEM) and Cell Counting Kit (CCK-8) were supplied by Thermo Fisher Scientific Inc. Leukocyte separation kit was purchased from Shanghai Yanjin Biotechnology Co., LTD.

### Preparation of Gau@TA/Thr gauze

The commercially available gauze (2 g) was immersed in a 30 mL solution of tannic acid (5%) and subjected to oscillating incubation at 37 °C for 48 h. The gauze was then removed and washed repeatedly with physiological saline solution for three times, then it was freeze-dried to obtain the tannic acid-treated gauze (Gau@TA). Next, 1 g of gauze was redispersed in 5 mL of the thrombin HEPES buffer solution (10 mM HEPES, pH = 7, the activity of thrombin in the buffer solution is 10 U/mL and 20 U/mL, respectively). After shaking at 4 °C for 2 h, the gauze was removed and washed repeatedly with buffer solution to remove the residual protein. Finally, the Gau@TA/Thr5 (1 g of gauze immobilized with 50 U of thrombin) and Gau@TA/Thr10 (1 g of gauze immobilized with 100 U of thrombin) gauze was freeze-dried and stored at − 80 °C for further use. Additionally, sample of 1 g gauze mixed with 50 U thrombin were also prepared and named as Gau/Thr.

### Characterization of the gauze

The chemical structures of Gau, Gau@TA and Gau@TA/Thr5 were confirmed by the Fourier-Transform Infrared Spectroscopy (FT-IR). All samples for FT-IR were treated by attenuated total reflectance (ATR) method. The morphologies of samples under different magnification were observed by scanning electronic microscopy (SEM).

### Thrombin activity

10 mg gauze sample was incubated with 180 μL HEPES buffer and 20 μL chromogenic substrate solution (4 mmol/L, S2302, HYPHEN BioMed) at 37 °C for 30 min. Then, the absorbance of the solution was measured immediately at 405 nm.

### Liquid absorption

All weighed gauze (*W*_*0*_) was immersed into water or rabbit citrated whole blood for a certain time. After that, the gauze was taken out and lightly dabbed on filter papers to remove the excess liquid, the weight of absorbent gauze was recorded as *W*_*1*_. The water or blood absorption ratio was calculated from the following equation:

$$ {\text{Liquid}}\;{\text{ absorption }}\;{\text{ratio}}\;(\% ) = \frac{{W_{1} - W_{0} }}{{W_{0} }} \times 100\% $$

### Water vapor permeability

A bottle containing 20 mL of distilled water was covered with the gauze sample. The mouth of the bottle was tightly sealed to avoid water vapor loss through the boundary. The entire system was incubated at 60 °C and the weight of bottle was measured at the certain time.

### Mechanical measurements

The mechanical tensile stress–strain evaluation was carried out by the uniaxial tensile test employing an Instron materials test system equipped with a 2 kN tension sensor at room temperature. All the gauze samples were prepared into stripes (120 mm in length × 50 mm in width × 200 μm in thickness) and stretched with a speed of 100 mm/min until break.

### Cytotoxicity assays

Firstly, the 0.5 g samples were incubated with 5 mL of DMEM culture medium for 24 h to prepare leaching liquor (0.1 g/mL). Then, the L929 fibroblast cells were seeded on 96-well plates and treated with the above leaching liquor. The fresh DMEM culture medium was set as the control group. Next, in vitro cytotoxicity assay was further performed by direct contact between the gauze and human dermal fibroblasts (HDFs), human umbilical vein endothelial cells (HUVECs). Before test, 5 mg samples were sterilized by ultraviolet irradiation. 500 μL HDFs and HUVECs with a density of 1 × 10^3^ cells were added onto samples in a 48-well plate and incubated for 24 h. According to the protocol of the CCK-8 kits, the absorbance of each well at 450 was tested (ABS_450_). The relative cell viability (%) was calculated using the following equation:

$$ {\text{Relative}}\;{\text{ cell }}\;{\text{viability}}\;(\% ) = \frac{{ABS_{450sample} }}{{ABS_{450control} }} \times 100\% $$

### Hemolysis assay

Briefly, 1 mL of anticoagulated whole blood collected from rabbit (All animal blood-related experiments were approved by Wenzhou Institute of UCAS) was mixed with 5 mL of a saline solution. Then, 10 mg sample was incubated with 1 mL of diluted whole blood at 37 °C for 4 h. Next, the above samples were centrifuged at 3000 rpm for 10 min to obtain liquid supernatant and the absorbance (ABS) of the liquid supernatant was measured at 540 nm. The absorbances at 540 nm of deionized water and saline solution were used as the positive control group and the negative control group, respectively. The hemolysis rate was calculated using the following equation:

$$ {\text{Hemolysis}}\;{\text{ ratio }}\;(\% ) = \frac{{ABS_{sample} - ABS_{negative} }}{{ABS_{positive} - ABS_{negative} }} \times 100\% $$

### Blood clotting in vitro

The whole blood clotting time (BCT) and blood clotting index (BCI) of all samples were detected. Firstly, 10 mg of gauze was incubated with 300 μL of citrated whole blood collected from rabbit/donor and 30 μL of CaCl_2_ (0.1 M) solution (pre-warmed at 37 °C for 30 min) in a 2 mL EP tube. Every 15 s, the EP tube was tilted to investigate the blood fluidity. The time was recorded when the blood clotting was formed. The test was cycled for three times. Next, 10 mg sample was incubated with 100 μL of citrated whole blood and 10 μL of CaCl_2_ (0.1 M) solution in a 50 mL EP tube. After incubation at 37 °C for 1 min and 3 min, 25 mL of deionized water was carefully added to the above tube and mixed at 20 rpm for 15 min to wash unclotted blood. According to the absorbance of red blood cells at 540 nm, the washed solution of each sample was measured (ABS_540 sample_). The absorbance of the citrated whole blood in deionized water was used for the control group (ABS_540 control_). The blood clotting index (BCI) was calculated using the following equation:

$$ {\text{Blood }}\;{\text{clotting }}\;{\text{index}}\;(\% ) = \frac{{ABS_{540sample} }}{{ABS_{540control} }} \times 100\% $$

### Red blood cells, platelets and leukocyte adhesion

Citrated whole blood collected from rabbit was centrifuged at 1000 rpm for 10 min to obtain platelet rich plasma (PRP) and red blood cells suspension. 5 mg of sample was placed into the 24-well plate and incubated with 200 μL of platelet-rich plasma (PRP) and diluted red blood cell suspension (diluted by a factor of 10) at 37 °C for 30 min. Then, the above samples were thoroughly washed with PBS (pH = 7.4) and treated with a 2.5% glutaraldehyde/PBS solution at 4 °C for 12 h. Lastly, gradient ethanol (55%, 65%, 75%, 85%, 95%, and 100%) was used to dehydrate the above samples before observing samples by SEM.

Besides, anticoagulated rabbit blood was used to obtain leukocyte suspension according to the method of the leukocyte separation kit. 5 mg of sample was placed into the 24-well plate and incubated with 100 μL of leukocyte suspension at 37 °C for 30 min. Then, the above samples were thoroughly washed with PBS (pH = 7.4) and treated with a 2.5% glutaraldehyde/PBS solution at 4 °C for 12 h. Lastly, gradient ethanol (55%, 65%, 75%, 85%, 95%, and 100%) was used to dehydrate the above samples before observing samples by SEM.

### Antibacterial property

The antimicrobial property of gauze was performed by colony counting. Gram-positive bacteria Staphylococcus aureus (*S. aureus* ATCC27217) and Gram-negative bacteria Escherichia coli (*E. coli* ATCC8099) were used as the model microorganisms. 10 mg of gauze was incubated with 900 μL sterilized PBS and 100 μL bacteria solution with the concentration of 5 × 10^5^ CFU/mL at 37 °C for 4 h. Afterwards, 100 μL bacterial suspension was incubated for 24 h on 1.5% LB agar plates and the total number of colonies was recorded. The bacteria solution treated without sample was used as blank control group. Three replicates were conducted for each group, and the survival ratio of bacteria was determined.

### Hemostatic efficacy in vivo

All animal experiments were performed in strict accordance with National Institutes of Health (NIH) guidelines for the care and use of laboratory animals, and the use of animals in this study was approved by the Wenzhou Institute of UCAS. We used SD rat liver volume defect, femoral artery, and carotid artery complete transection models to evaluate the hemostasis efficacy of all samples. Five rats with the weight of 350 g were used for each material in vivo test.

Hemostasis study in SD rat liver: the rat was anesthetized by intramuscular injection of quantitative Zoletil solution. Then, its abdominal hair was shaved off and abdominal cavity was opened to expose the left lobe liver, a pre-weighed filter was placed at the bottom of the liver and a piece of liver (2 cm) was cut off to cause bleeding. The pre-weighed gauze was applied to the wound to stop bleeding. The rats in the blank control group were treated without any materials. The hemostatic time and blood loss amount (blood absorbed by gauze and filter paper) in the operation were recorded.

Hemostasis study in SD rat femoral artery: the rat was anesthetized and its leg hair was shaved off. The overlying muscle of groin was opened to exposed femoral artery, and artery and vein were dissected and transected fully. Immediately, the pre-weighed gauze was applied on the wound with manual press. The blood loss amount was recorded.

### Subcutaneous implantation

The SD rat was anesthetized by intramuscular injection of quantitative Zoletil solution. The dorsal skin was opened using surgical scissors, and gauze was inserted subcutaneously, while no material was inserted in the blank control group. After completion, the dorsal wound was sutured, and the rats were continued to be housed. One week later, the skin from implantation site was removed and fixed in 2.5% glutaraldehyde solution. Then, the tissues were dehydrated in different concentrations of ethanol and embedded in paraffin. Paraffin blocks were sectioned consecutively, and tissue sections were placed on slides. Subsequently, the sections were deparaffinized in xylene, stained with hematoxylin and eosin. Finally, the sections were scanned using a slide scanner.

### Statistic analysis

Unless otherwise specified, all experiments have been conducted a minimum of three times to ensure accuracy and reliability. The data are represented as mean ± standard deviation and were compared through the one-way ANOVA using SPSS software. If the p-value is less than 0.05, it indicates a significant difference between the groups. This rigorous statistical analysis helps to ensure the robustness and validity of the findings.

### Statement

Animal experimental protocols were approved by the Institutional Animal Ethics Committee for Experimentation on Animals of Wenzhou Institute of UCAS and followed the National Institutes of Health Guide for the Care and Use of Laboratory Animals.

### Ethics declarations

All experiments were performed in accordance with relevant ARRIVE guidelines and regulations.

### Approval for animal/clinical experiments

All animal experiments were approved by the Institutional Animal Care and Use Committee of Wenzhou Institute of UCAS (WIUCAS23030133). All clinical experiments were approved by the Ethics Committee of Municipal Hospital affiliated to Taizhou University (LWYJ2024187).

## Results and discussion

### Preparation of Gau@TA/Thr

**Figure 1:**
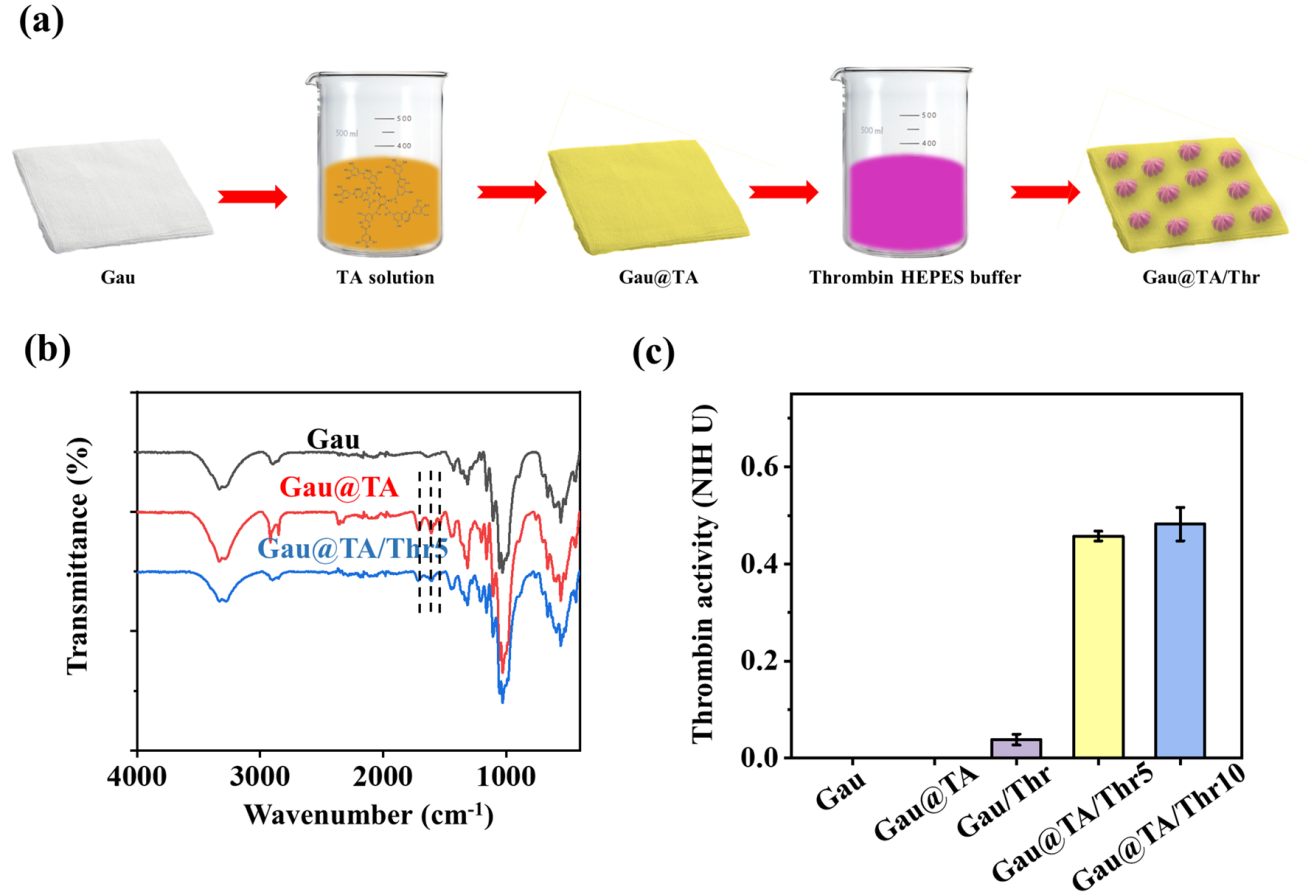
(**a**) Synthetic scheme of thrombin immobilized gauze. (**b**) FT-IR spectra of Gau, Gau@TA and Gau@TA/Thr5. (**c**) Thrombin activity of all samples.

It was reported that TA could construct layer-by-layer self-assembly films by utilizing its versatile binding capabilities^25,26^. Furthermore, due to its multiple interaction mechanisms, proteins can bind effectively to the TA-modified surface and retain their activity. In this work, the preparation process of the Gau@TA/Thr gauze was shown in Fig. 1a. After 48 h treatment of TA solution (5% w/v), the commercial gauze was fixed with a layer of tannic acid coating (Gau@TA), causing its color to change from white to light yellow. With the anchor moieties (catechol and pyrogallol) of tannic acid, thrombin was further immobilized onto the gauze. After freeze-drying, the Gau@TA/Thr5 and Gau@TA/Thr10 gauzes were obtained. The chemical structure of the Gau, Gau@TA and Gau@TA/Thr5 was confirmed through the FT-IR spectroscopy. As shown in Fig. 1b, the stretching vibrations of C=O (carboxylic ester) groups of TA was appeared at 1704 cm^−1^ in the spectrum of Gau@TA and Gau@TA/Thr5. The peaks at 1607 cm^−1^ and 1526 cm^−1^ were consistent with aromatic C–C stretches, which also belong to TA^27,28^. It was indicated that the TA was successfully coated onto the gauze. Besides, the peak corresponding to the amide bond stretching vibration of thrombin was not found in the spectrum of the Gau@TA/Thr5, which may result from the low concentration of the thrombin content.

To demonstrate that thrombin has been firmly immobilized onto the gauze surface, we tested the enzyme activity of the samples using a thrombin chromogenic substrate. It was worth noting that the thrombin loaded gauze (Gau/Thr) prepared by impregnation and washing process was also tested. As shown in Fig. 1c, the results showed that there was no thrombin activity in the group of Gau and Gau@TA. The 10 mg of Gau/Thr, Gau@TA/Thr5 and Gau@TA/Thr10 exhibited thrombin activity of 0.038 ± 0.01, 0.457 ± 0.01, and 0.4821 ± 0.03 U, respectively. From the results, it is evident that more than 90% of the thrombin has been effectively immobilized on the surface of the Gau@TA/Thr5. In contrast, the immobilization efficiency of Gau@TA/Thr10 is somewhat lower, possibly due to the limited content of tannic acid in the gauze. Based on the results of coagulation activity and immobilization efficiency, we have chosen Gau@TA/Thr5 as the experimental group for subsequent experiments. Furthermore, compared to conventional impregnation methods, this tannic acid immobilization method better preserves the content and activity of thrombin. This also ensures that the thrombin in the gauze may not easily release into the blood during use, thereby reducing the risk of thrombosis. Compared to directly spraying large amounts of thrombin drugs onto wounds, it is theoretically safe to use Gau@TA/Thr as it is unlikely for thrombin to enter the bloodstream. As for the stability of thrombin on the gauze, considering the activity of thrombin in the composite gauze and its dry state, its shelf life theoretically should not be lower than that of commercially available lyophilized thrombin powder, which remains stable when stored at 2–8 °C for 12 months and at room temperature for 30 days, with a requirement to be used within 12 h of opening. Such storage conditions also ensure that the material can be used as a prehospital emergency product.

### Characterization of Gau@TA/Thr

**Figure 2:**
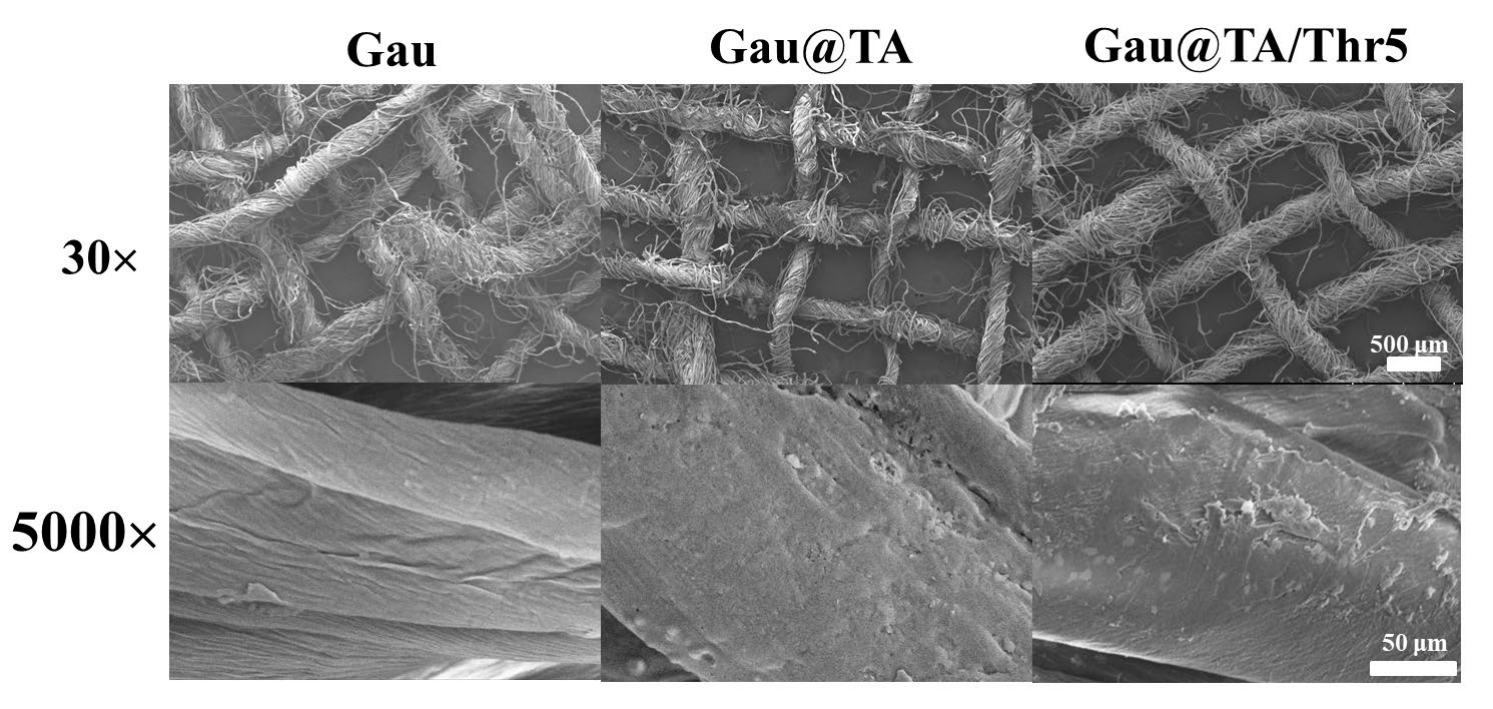
SEM images of three gauzes in 30 and 5000 magnifications.

The morphology of three gauzes were studied and the SEM images at low magnification showed that Gau, Gau@TA and Gau@TA/Thr5 had porous structures, which was conducive to concentrate blood and absorbing wound exudate (Fig. 2). Furthermore, at a large magnification, the Gau@TA and Gau@TA/Thr5 gauze surface had a layer of polymer structure, which may be due to the TA coating. The experimental results indicated that the introduction of TA did not disrupt the porous structure of the gauze but merely formed a coating on the surface of the gauze.

**Figure 3:**
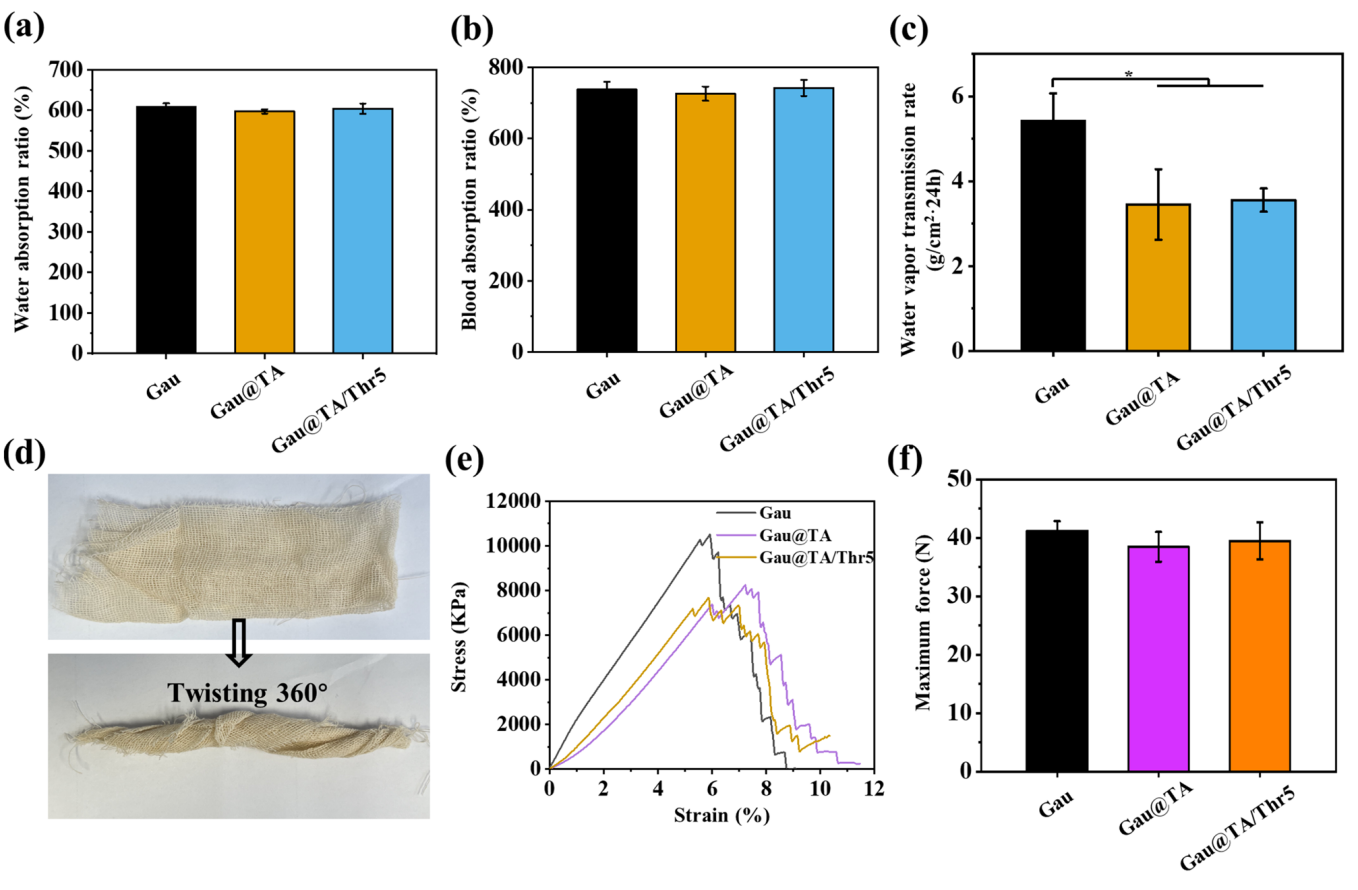
(**a**) The maximum water and (**b**) maximum blood absorption ratio of gauzes. (**c**) The water vapor transmission rate of gauzes. (*p < 0.05). (**d**) Photographs of Gau@TA/Thr5 in situ twisting. (**e**) The tensile stress–strain curves of gauzes. (**f**) The maximum force of gauzes. All data in panels (**a**–**e**) are shown as mean ± SD (n = 3).

The well liquid absorptive capacity was one of the basic performances of hemostatic materials, as it facilitated the enrichment of effective components in the blood, thereby accelerating the coagulation process^29^. The maximum water absorption ratio of the three gauze was presented in Fig. 3a, the value of Gau, Gau@TA and Gau@TA/Thr5 was 608 ± 9%, 597 ± 5% and 604 ± 13%, respectively. Furthermore, the maximum blood absorption ratio of Gau, Gau@TA and Gau@TA/Thr5 was 738 ± 22%, 726 ± 20% and 742 ± 22% (Fig. 3b). The experimental results indicated that there was virtually no difference in the maximum fluid absorption ratio between commercial gauze and modified gauze. As a result, the outstanding fluid-absorbing performance would contribute to the gauze's ability to address acute hemorrhage wounds. In addition to hemostasis, gauze is frequently utilized for wound care. So, we also tested the water vapor transmission of gauze. The value of Gau, Gau@TA and Gau@TA/Thr5 group was 5.4 ± 0.6, 3.4 ± 0.8 and 3.6 ± 0.3 g/cm^2^·24 h, respectively (Fig. 3c). It was found that the breathability of gauze was decreased after treatment with TA. Combining the results of SEM images, our analysis suggested that the diminished breathability of the gauze may be attributed to the presence of a polymer coating on its surface. This coating may undergo swelling upon contact with water vapor, which hindered the passage of water vapor.

The excellent mechanical performance would facilitate gauze in addressing various bleeding wounds. Firstly, the flexibility of gauze was displayed in Fig. 3d, the TA coating and thrombin immobilization on the gauze did not affect its flexibility (easily twisted 360° in situ), which allows for great potential for hemostasis on different tissue surfaces. Next, the tensile stress–strain curve was performed to further evaluate the mechanical properties of the gauze. As shown in Fig. 3e,f, it was observed that the maximum tensile force of Gau@TA and Gau@TA/Thr5 was reduced, possibly due to prolonged immersion in the solution during the preparation process. However, there was no significant difference in the maximum force values exhibited by Gau, Gau@TA and Gau@TA/Thr5. In summary, the modification treatment applied to the gauze did not have a significant impact on its mechanical performance.

### Blood clotting in vitro

**Figure 4:**
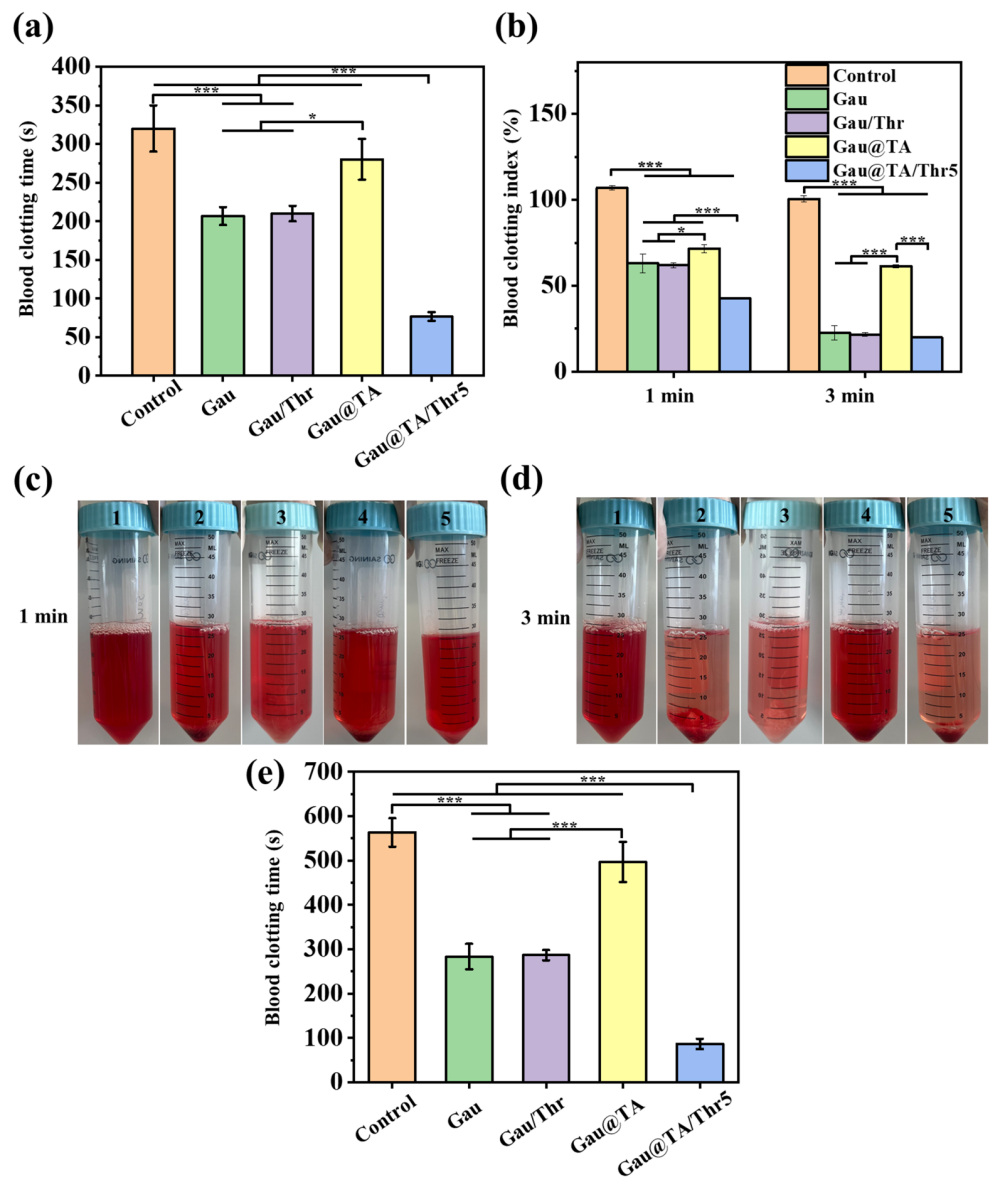
(**a**) The rabbit whole blood clotting time of all samples. (**b**) The rabbit whole blood clotting index of all samples. (**c**) Photographs showing BCI assays of all samples at 1 min (1: control, 2: Gau, 3: Gau/Thr, 4: Gau@TA, 5: Gau@TA/Thr5). (**d**) Photographs showing BCI assays of all samples at 3 min (1: control, 2: Gau, 3: Gau/Thr, 4: Gau@TA, 5: Gau@TA/Thr5). (**e**) The human whole blood clotting time of all samples. (*p < 0.05, ***p < 0.001).

Firstly, the blood clotting time was evaluated in vitro according to the mobility of blood. As shown in Fig. 4a, the results showed that the blood clotting time of whole blood without any treatment was 320 ± 30 s. The commercial gauze and Gau/Thr took 207 ± 12 s and 210 ± 10 s to complete blood clotting, which was shorter than the blank control group. The procoagulant effect may be attributed to the inherent properties of the gauze material itself. However, the clotting time of Gau@TA was extended to 280 ± 16 s. It is possible that the coating of TA may have affected the surface characteristics of the gauze. After immobilized with thrombin, the Gau@TA/Thr5 could accomplish coagulation within 77 ± 6 s. It significantly reduced the clotting time comparing to the other groups. Notably, in the Gau/Thr group, thrombin was difficult to retain effectively on the gauze. As a result, the in vitro coagulation effect of Gau/Thr was significantly lower than that of Gau@TA/Thr5.

Next, the blood clotting ability of all samples were evaluated using BCI. The hemoglobin solution was collected after rinsing the whole blood incubated sponge. As shown in Fig. 4b–d, the BCI of Gau, Gau/Thr, Gau@TA and Gau@TA/Thr5 was 63.1 ± 5.5%, 61.9 ± 1.3%, 71.6 ± 2.6% and 42.7 ± 0.4% after 1 min incubation. The value of 3 min in Gau, Gau/Thr, Gau@TA and Gau@TA/Thr5 group reached to 22.6 ± 4.1%, 21.7 ± 1.1%, 61.4 ± 1.0% and 19.9 ± 0.1%, respectively. The experimental results indicated that gauze immobilized with thrombin exhibited an outstanding blood clotting capacity. Upon contact with blood, the gauze could rapidly concentrate clotting proteins and red blood cells. In the presence of thrombin, a stable fibrin clot is quickly formed. Besides, the coagulation performance of Gau@TA appeared to be inferior, possibly associated with its prolonged coagulation time.

Furthermore, we incubated the materials with blood samples from donors to evaluate the procoagulant ability of the gauzes in a more relevant environment. As shown in the Fig. 4e, the in vitro clotting times for control, Gau, Gau/Thr, Gau@TA and Gau@TA/Thr5 group was 563 ± 32 s, 283 ± 28 s, 287 ± 12 s, 497 ± 45 s and 87 ± 12 s, with the Gau@TA/Thr5 still exhibiting the shortest in vitro clotting time.

### RBCs, platelets and leukocytes adhesion

**Figure 5:**
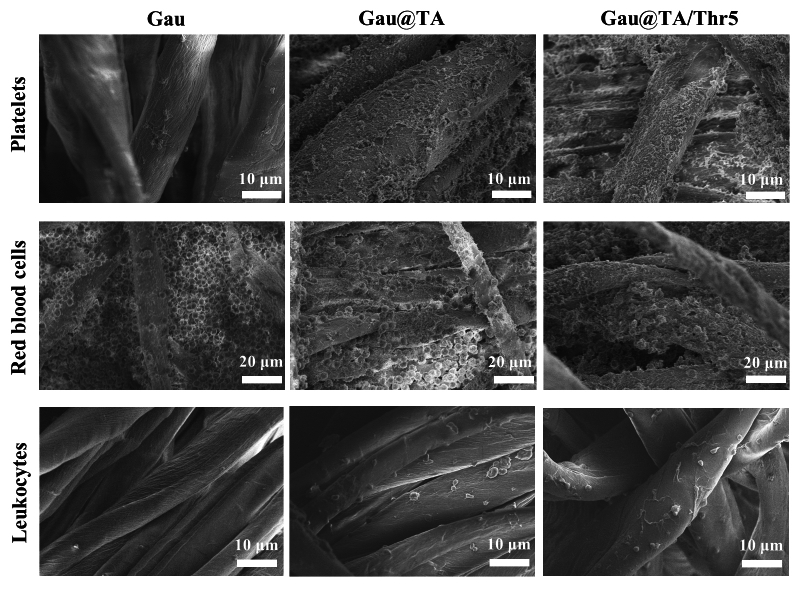
The SEM images showing the platelets, red blood cells and leukocytes adhered to the surface of gauzes.

The main components of blood, platelets, and red blood cells, have been reported to play crucial roles in hemostasis. An increase in the number of red blood cells was found to contribute to thrombosis. Furthermore, the aggregation of red blood cells could effectively seal off wounds, promoting hemostasis. It was reported that platelets were involved in the primary hemostatic process. When blood vessels were damaged, the platelets were activated and aggregate to form the primary clot^16,30^. Therefore, we investigated the impact of TA coating and thrombin on the adhesion of red blood cells and platelets to gauze. As shown in Fig. 5, it was found that there were small amounts of platelets adhering to the surface of the blank gauze. In contrast, a significant quantity of platelets, along with proteins, adhered to the surface of the Gau@TA and Gau@TA/Thr5. This may be attributed to tannic acid (TA) on the gauze surface, which bound to relevant proteins and subsequently associated with platelets. Furthermore, all Gau, Gau@TA and Gau@TA/Thr5 could attract the red blood cells. In particular, the cells tended to gather on the pore structure of the Gau, while the cells could also adhere to the surface of the Gau@TA and Gau@TA/Thr5. In summary, we considered that the binding of TA enhanced the adhesion capacity of gauze to proteins, platelets, and red blood cells in the blood. Simultaneously, under the catalytic activity of thrombin within the gauze, the fibrinogen rapidly cross-linked to form a stable blood clot.

Besides, it was well know that leukocytes (particularly neutrophils and monocytes) also contributed to the clotting process. Moreover, leukocytes were central cells that mediated the host's inflammatory and immune responses. Understanding the interaction between leukocytes and the material could exclude any inflammatory reaction induced by the gauze in contact with blood. As shown in Fig. 5, it was found that leukocytes also tended to adhere to Gau@TA and Gau@TA/Thr5. Although this result was more favorable for the hemostatic promotion of TA-coated gauze, the adhesion and aggregation of leukocytes may lead to an increased degree of inflammation. Of course, considering that gauze is not a long-term implant material like vascular stents, short-term inflammation may not have a significant impact. Besides, TA could also exert anti-inflammatory effects through other mechanisms, thereby alleviating this issue^25^.

### The antibacterial property

During the first aid process, the bleeding wound is exposed to the external environment, making it susceptible to bacterial infection, which can result in greater difficulty in subsequent wound care. Hence, to protect wound tissue from external bacterial infection, an ideal hemostatic agent should possess inherent antibacterial properties. TA was considered as an antimicrobial agent, and was used as a therapy of choice for the clinical treatment of burns in the mid-1920s. It was reported that TA inhibited the surface colonization of *S. aureus* via a mechanism dependent upon the putative staphylococcal antigen A^27^.

**Figure 6:**
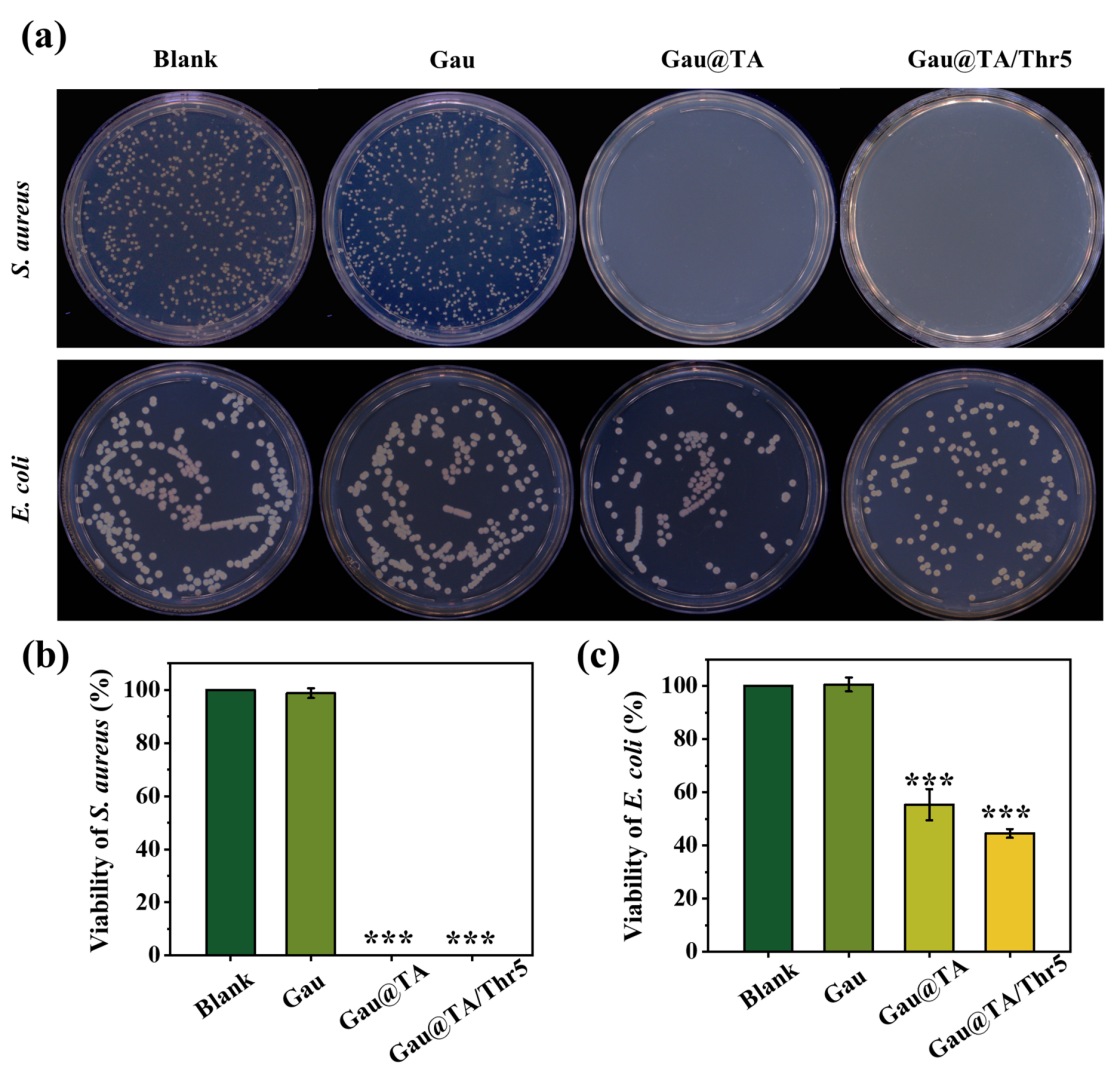
Antibacterial property all samples. (**a**) Representative images showing the survival bacterial in the culture plate. Quantitative antibacterial test results of different gauze to (**b**) *S. aureus*, and (**c**) *E. coli* (***p < 0.001).

The antibacterial properties against *S. aureus* and *E. coli* of all gauzes were evaluated using agar plate method (Fig. 6a–c). After co-culturing bacteria with gauze, we found that compared to the control group, the bacterial count in the Gau group remained largely unchanged, indicating that conventional gauze lacks any antibacterial properties. However, after coating with TA, the bacterial count significantly decreased in Gau@TA and Gau@TA/Thr5 group, particularly against *S. aureus*, with almost no bacterial growth observed. As confirmed by statistical analysis, TA coated gauzes demonstrated an obvious antibacterial effect for gram-positive *S. aureus*, and the bacterial viability of the modified gauze was nearly 0. Besides, the Gau@TA and Gau@TA/Thr5 also showed antibacterial effect against gram-negative *E. coli*, with the bacteria viability of 55.3 ± 5.8% and 44.5 ± 1.6%, respectively. It is worth noting that, considering the adhesive properties of TA, there may be a slight margin of error in the bacterial count process.

### In vivo hemostatic performance

Uncontrolled bleeding, especially from extensive organ damage or rupture of the carotid artery, constitutes a primary cause of death in many trauma cases. Effective hemostatic intervention can prolong the window for treatment and enhance the chances of patient survival. Conventional hemostatic materials are not effective for uncontrolled hemorrhage. In this work, thrombin immobilized gauze was prepared. The liver volume defect and femoral/carotid artery injury of SD rats were implemented to evaluate the hemostatic performance of different gauzes.

**Figure 7:**
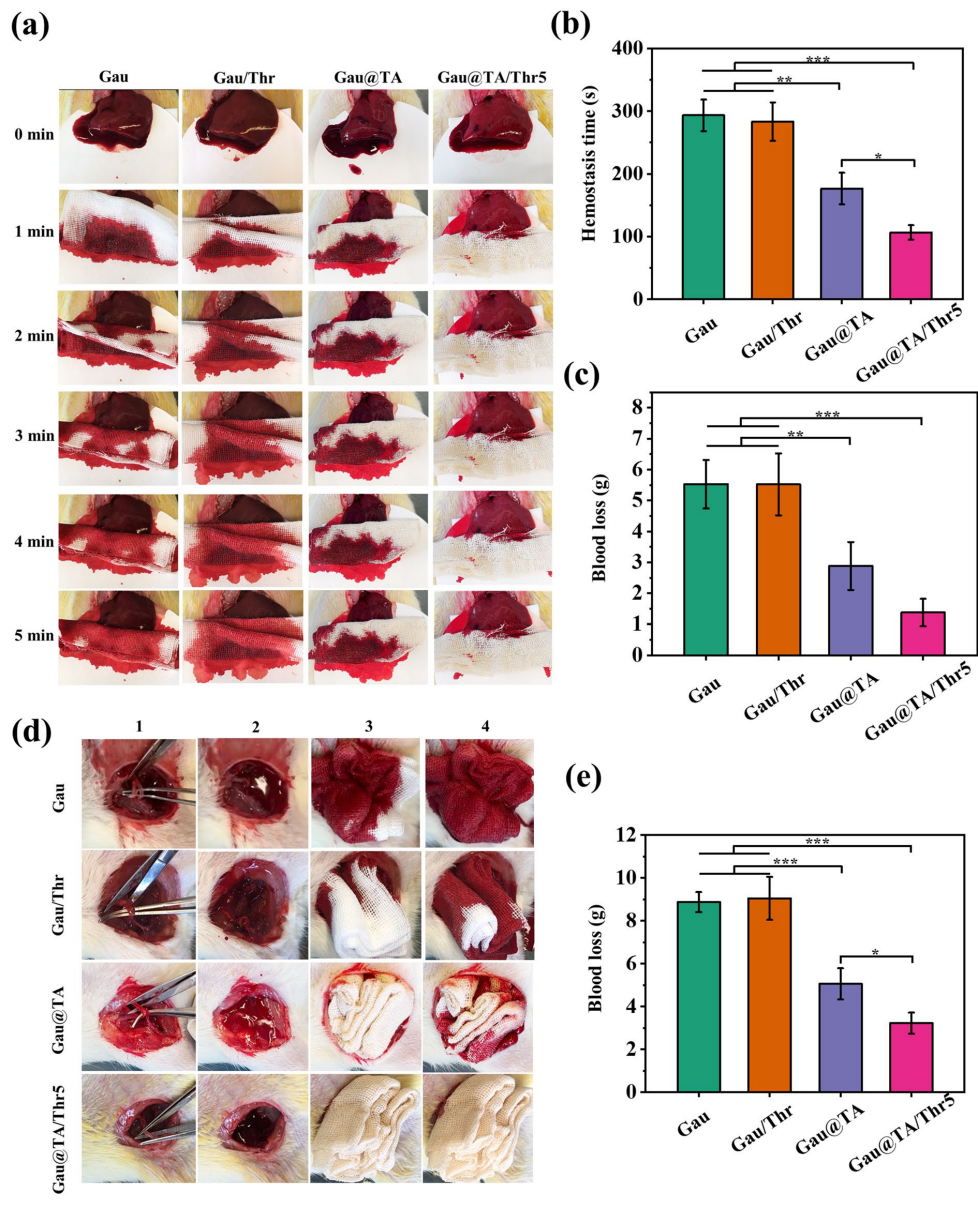
In vivo hemostatic evaluation of gauze using SD rats. (**a**) Photographs of the bleeding and hemostasis process within 5 min. (**b**) The hemostasis time of all gauzes in rat liver injury. (**c**) The blood loss of all gauzes in rat liver injury. (**d**) Operational process of SD rat femoral artery hemostasis (1: femoral artery was exposed, 2: hemorrhage was created, 3: gauze was applied, 4: complete hemostasis was achieved). (**e**) Blood loss of all samples in rat femoral artery injury (*p < 0.05, **p < 0.01, ***p < 0.001).

In a liver bleeding model, a portion of the tissue was excised to induce massive hemorrhage, and gauze was applied to the bleeding wound. The hemostasis time and the amount of bleeding was recorded during the experimental process. As shown in Fig. 7a–c. The statistical results showed that hemostatic time in Gau, Gau/Thr, Gau@TA and Gau@TA/Thr5 group was 293 ± 25 s, 283 ± 30 s, 177 ± 25 s and 107 ± 12 s, respectively. The blood loss of Gau, Gau/Thr, Gau@TA and Gau@TA/Thr5 group was 5.5 ± 0.8 g, 5.5 ± 1.0 g, 2.9 ± 0.8 g and 1.4 ± 0.4 g, respectively. Compared to commercially available gauze and Gau/Thr, Gau@TA and Gau@TA/Thr5 gauzes could reduce clotting time and blood loss in vivo. Due to the difficulty of immobilizing thrombin directly on the surface of gauze, the in vivo hemostatic performance of Gau/Thr showed no significant difference compared to commercia gauze. The favorable hemostatic performance of tannic acid-coated gauze may be attributed to tannic acid's ability to adhere to plasma proteins upon contact with blood, thereby block off bleeding from wounds. Furthermore, it was observed that Gau@TA/Thr5 gauze exhibited a significantly enhanced hemostatic performance during the experimental process.

To increase the amount of bleeding, we established a SD rat femoral artery transection hemorrhage model (Fig. 7d). As shown in Fig. 7e, in Gau, Gau/Thr and Gau@TA group, the total blood loss was 8.9 ± 0.5 g, 9.0 ± 1.0 g and 5.0 ± 0.7 g. TA coating gauze had reduced the blood loss. Gau@TA/Thr5 led to the least blood loss (3.2 ± 0.5 g). The Gau@TA/Thr5 group significantly reduced the amount of bleeding from the femoral artery rupture, thereby extending the treatment time.

Similarly, reports on thrombin-composite gauze also exist. For instance, Liu et al. reported a hemostatic gauze material by promoting in situ thrombin induction on the surface of gauze. However, the hemostatic effect of this sponge was insufficient, only comparable to commercially available oxidized cellulose gauze^31^. Shi et al. synthesized a series of ion/thrombin hybrid microflowers and thrombin/MOF materials to enhance the stability of thrombin and loaded them onto gauze. While the composite gauze retained thrombin activity and improved its storage stability. However, this preparation process is relatively complex, and the material lacks verification of its hemostatic effect in large bleeding models^32^. In this study, we applied TA coating to the surface of gauze to obtain an adhesive layer. This layer not only adheres the components in the blood, such as proteins, platelets, and red blood cells, but also serves as a medium to load the active component (thrombin) efficiently and safely. Consequently, it enhances the procoagulant activity of the gauze. Through the synergistic hemostatic mechanisms of the gauze, even in the management of large and rapidly blood flowing wounds, a significant reduction in hemostatic time and blood loss can be achieved. This multi-faceted hemostatic effect would provide valuable time for subsequent medical interventions. Moreover, the use of TA also imparts inherent antibacterial properties to the gauze, thereby protecting wound tissue from external bacterial infection. Additionally, in recent years, some cryogels with expanding properties have been prepared for controlling acute bleeding and non-compressible bleeding^33–35^. Compared to such materials, Gau@TA/Thr5 exhibits superior procoagulant activity, and its flexibility makes it more suitable for the management of surface wounds. However, its effectiveness in managing deep, irregular, and non-compressible wound bleeding is often inferior to that of cryogels. Furthermore, thrombin-composite gauze carries the risk of immunogenicity compared to other novel polymer-based hemostatic agents.

### Biocompatibility

**Figure 8:**
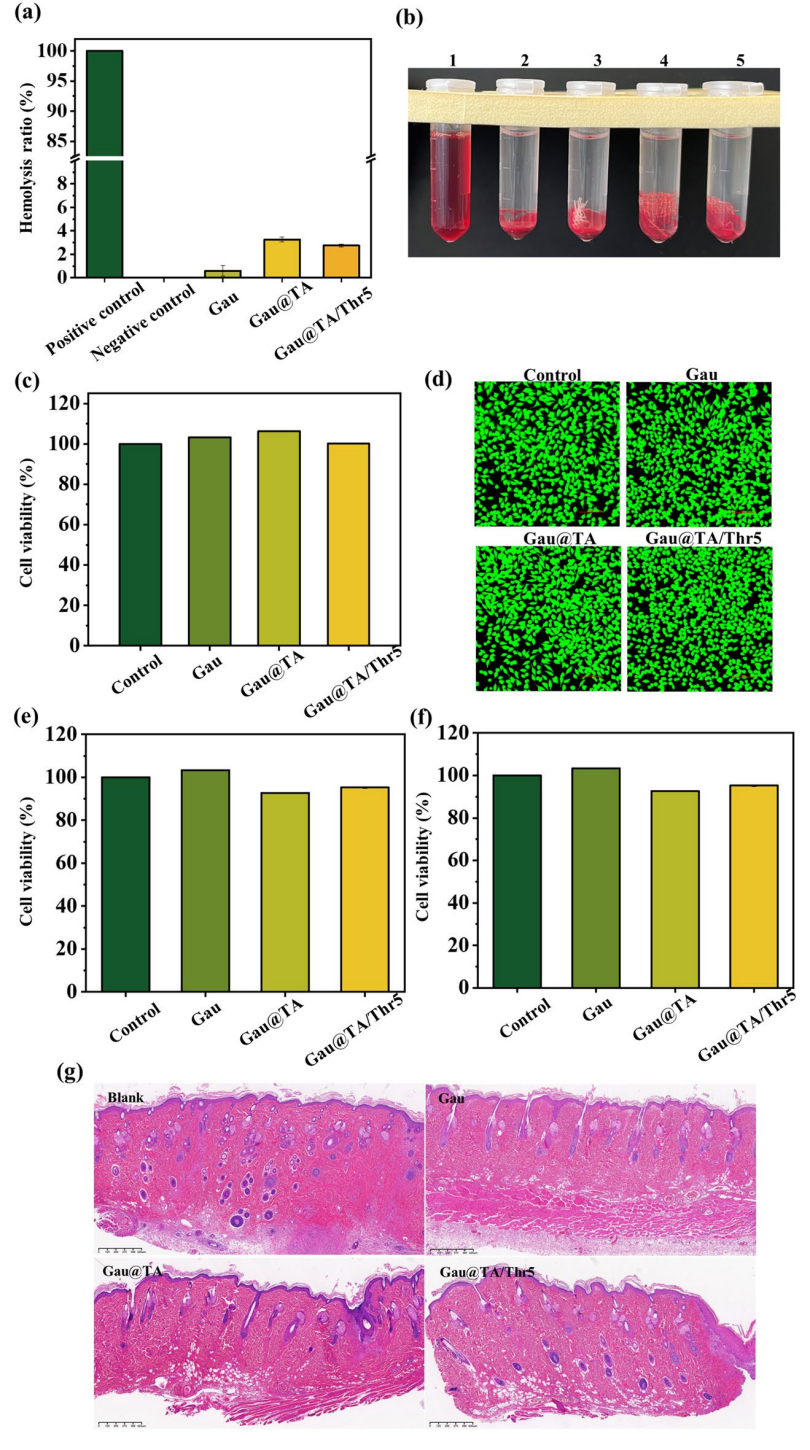
(**a**) Hemolysis ratio for all samples. (**b**) Photographs showing the hemolysis assays (1: deionized water (DW), 2: normal saline (NS), 3: Gau, 4: Gau@TA, 5: Gau@TA/Thr5). (**c**) In vitro cell viability of L929 cells treated with sample extracts. (**d**) LIVE/DEAD staining images of L929 cells treated with sample extracts. In vitro cell viability of (**e**) HDFs and (**f**) HUVECs directly cultured with samples. (**g**) Hematoxylin–Eosin (H&E) staining of subcutaneous implanted skin tissue. Scar bar: 625 µm.

Hemolysis assay in vitro was adopted to assess the hemocompatibility of materials. Citrated whole blood treated with suspension solutions of Gau, and Gau@TA and Gau@TA/Thr5 only had a hemolysis percentage of 0.6 ± 0.43%, 3.3 ± 0.20% and 2.8 ± 0.12%, which were within the permissible range of biomaterials (Fig. 8a,b). Next, in vitro cytocompatibility of gauze was investigated by CCK-8 assay and fluorescence staining. As shown in Fig. 8c, the relative cell viability of all sample extract was above 95%, at a concentration of 1 mg/mL. It was indicated that these gauze extracts were noncytotoxic after incubated with L929 fibroblast cells for 24 h. The cell morphology was photographed to display the L929 cells condition and the stained images further confirmed that no significant difference was observed between control cells and cells treated with samples (Fig. 8d). Furthermore, as shown in Fig. 8e,f, it was clear to see that the gauzes were still biocompatible after directly incubated with HDFs and HUVECs for 24 h. At last, gauzes were implanted subcutaneously for evaluating its in vivo safety. Pathological images demonstrated that, compared to the blank control group, both commercial gauze and modified gauze did not exhibit significant inflammatory reactions in the skin tissue, indicating good biocompatibility in vivo (Fig. 8g). These results from hemolytic, cytotoxic, and H&E staining tests indicated that the Gau@TA and Gau@TA/Thr5 had excellent hemocompatibility, cytocompatibility, and histocompatibility.

## Conclusion

In this work, the procoagulant components-thrombin was successfully immobilized on the surface of cellulose gauze by utilizing the phenolic hydroxyl group and hydrophobic segments of tannic acid (TA). The modified gauze (Gau@TA/Thr5) not only retained the activity of thrombin but also demonstrated excellent fluid-absorbing capacity, breathability, flexibility, mechanical strength, and biocompatibility. In in vitro clotting experiments, the Gau@TA/Thr5 demonstrated significantly enhanced procoagulant activity due to the efficient immobilization of thrombin, leading to a halving of the coagulation time compared to the commercial gauze. Furthermore, the gauze also exhibited outstanding hemostatic performance in multiple uncontrollable hemorrhage models in SD rats, thereby extending the critical treatment window in emergency situations. As the result, the method of TA coating provides a safe and efficient strategy for immobilizing thrombin onto polymeric hemostatic materials. The Gau@TA/Thr5 gauze will be a promising hemostatic material for prehospital and clinical massive hemorrhage control.

## Data Availability

Data will be made available on request. Data can be requested from corresponding author (dr.zhengcheng@foxmail.com).
